# Greater Consideration of Animals Will Enhance Coastal Restoration Outcomes

**DOI:** 10.1093/biosci/biac088

**Published:** 2022-10-12

**Authors:** Michael Sievers, Christopher J Brown, Christina A Buelow, Robin Hale, Andria Ostrowski, Megan I Saunders, Brian R Silliman, Stephen E Swearer, Mischa P Turschwell, Stephanie R Valdez, Rod M Connolly

**Affiliations:** Coastal and Marine Research Centre, Australian Rivers Institute, and with the School of Environment and Science, Griffith University, Gold Coast, Queensland, Australia; Coastal and Marine Research Centre, Australian Rivers Institute, and with the School of Environment and Science, Griffith University, Gold Coast, Queensland, Australia; Coastal and Marine Research Centre, Australian Rivers Institute, and with the School of Environment and Science, Griffith University, Gold Coast, Queensland, Australia; Arthur Rylah Institute for Environmental Research, Department of Environment, Land, Water, and Planning, Heidelberg, Victoria, Australia; Coastal and Marine Research Centre, Australian Rivers Institute, and with the School of Environment and Science, Griffith University, Gold Coast, Queensland, Australia; CSIRO Oceans and Atmosphere, St Lucia, Brisbane, Australia; Nicholas School of the Environment, Duke University, Beaufort, North Carolina, United States; National Centre for Coasts and Climate and with the School of Biosciences, University of Melbourne, Parkville, Victoria, Australia; Coastal and Marine Research Centre, Australian Rivers Institute, and with the School of Environment and Science, Griffith University, Gold Coast, Queensland, Australia; Nicholas School of the Environment, Duke University, Beaufort, North Carolina, United States; Coastal and Marine Research Centre, Australian Rivers Institute, and with the School of Environment and Science, Griffith University, Gold Coast, Queensland, Australia

**Keywords:** decision science, ecological restoration, rehabilitation, translocation, transplantation

## Abstract

As efforts to restore coastal habitats accelerate, it is critical that investments are targeted to most effectively mitigate and reverse habitat loss and its impacts on biodiversity. One likely but largely overlooked impediment to effective restoration of habitat-forming organisms is failing to explicitly consider non-habitat-forming animals in restoration planning, implementation, and monitoring. These animals can greatly enhance or degrade ecosystem function, persistence, and resilience. Bivalves, for instance, can reduce sulfide stress in seagrass habitats and increase drought tolerance of saltmarsh vegetation, whereas megaherbivores can detrimentally overgraze seagrass or improve seagrass seed germination, depending on the context. Therefore, understanding when, why, and how to directly manipulate or support animals can enhance coastal restoration outcomes. In support of this expanded restoration approach, we provide a conceptual framework, incorporating lessons from structured decision-making, and describe potential actions that could lead to better restoration outcomes using case studies to illustrate practical approaches.

Restoration is a key challenge of the twenty-first  century, because ecosystems are being increasingly lost and degraded (McDonald et al. 2016, Gann et al. 2019, Halpern et al. 2019, He and Silliman 2019, Williams et al. 2021). Conservation efforts have traditionally been focused on the protection of intact habitats or the mitigation of stressors, but these approaches have failed at times to reverse widespread trends in ecological degradation (Lotze et al. 2011, Díaz et al. 2019, Griffiths et al. 2020). The restoration of coastal and marine ecosystems is particularly important, because over 775 million people depend on coastal systems; they have a relatively high role in climate mitigation and adaptation, and; have undergone widespread loss (Duarte et al. 2013a, Selig et al. 2019, Dunic et al. 2021, Murray et al. 2022). Restoration is therefore necessary to reverse coastal habitat loss and degradation, enhance biodiversity, and reestablish ecosystem services such as fisheries production, coastline protection, and climate change mitigation (Wood et al. 2019, Abelson et al. 2020, Waltham et al. 2020, Buelow et al. 2022). Coastal restoration efforts are consequently accelerating, supported by international calls to action, including the UN Decade of Ecosystem Restoration and Sustainable Development Goals (Perring et al. 2015, Young and Schwartz 2019, Sheaves et al. 2021). However, despite some notable exceptions (Saunders et al. 2020), coastal restoration projects tend to be small scale and expensive and have low survival rates of the habitat-forming species (Dale et al. 2014, Bayraktarov et al. 2016, van Katwijk et al. 2016, Cooke et al. 2019).

We posit that an often overlooked but ecologically significant gap in the implementation of restoration is that non-habitat-forming animals are not explicitly and holistically included in restoration planning, implementation, and monitoring (Halpern et al. 2007, Jones and Davidson 2016, Hale et al. 2019). Only 13% of the studies in a review of priorities and motivations of marine coastal restoration research, for instance, measured non-habitat-forming animal responses (Bayraktarov et al. 2020), and only a small proportion of seagrass restoration efforts explicitly manipulate animals (Zhang et al. 2021). In the present article, habitat-forming animals are those that form the structural habitat being restored, such as reef building corals and oysters, whereas non-habitat-forming animals are all other animals. Although the detrimental impacts that animals can have on restored habitats are being considered in some specific restoration efforts (e.g., in those that remove species that predate or graze on transplanted habitat formers), many animals perform a suite of vital functions that are necessary for ecosystem persistence, enhance ecosystem resilience through assisting disturbance recovery, and drive many of the services that restoration actively seeks to enhance (figure 1; Halpern et al. 2007). Consideration of mutualisms with animals that enhance habitat-forming species growth and survival is particularly important for coastal habitats that are frequently disturbed by natural and anthropogenic sources that make restoration inherently difficult (Lewis and Anderson 2012, Renzi et al. 2019, Gagnon et al. 2020). One of the most well-known mutualisms likely to have significant benefits for coastal restoration initiatives exists between bivalves and coastal vegetation. Bivalves, for example, can reduce sulfide stress in seagrass and mangroves and can facilitate saltmarsh vegetation by providing nutrients and reducing erosion (figure 1; also see Gagnon et al. 2020 and the references within).

**Figure 1.**
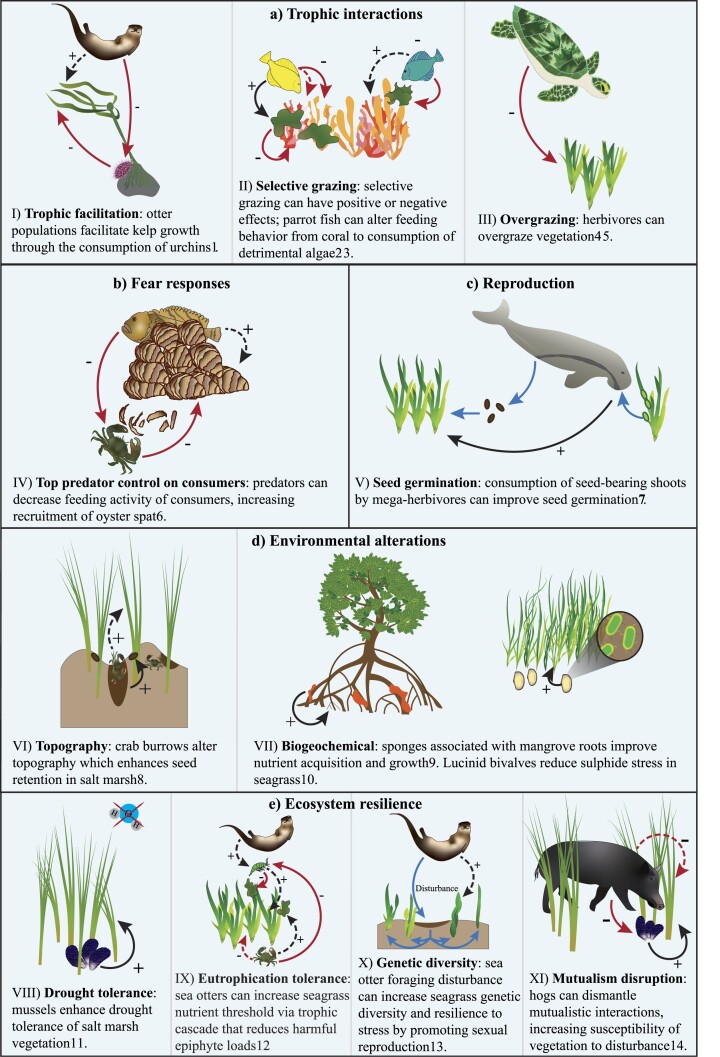
Conceptual diagram depicting positive (black arrows) and negative (red arrows), indirect (dashed lines) and direct (solid lines) interactions (with respect to effects on the habitat-forming species) between non-habitat-forming animals and coastal habitats. The sets of blue arrows indicate a context dependent pathway of effect. Interactions are categorized as (a) trophic interactions, (b) fear responses, (c) reproduction, (d) environmental alterations, and (e) ecosystem resilience. References: ^1^Eger et al. 2020, ^2^Rotjan and Lewis 2006, ^3^Seraphim et al. 2020, ^4^Christianen et al. 2014, ^5^Gangal et al. 2021, ^6^Grabowski 2004, ^7^Tol et al. 2021, ^8^Qiu et al. 2021, ^9^Ellison et al. 1996, ^10^van der Heide et al. 2012, ^11^Angelini et al. 2016, ^12^Hughes et al. 2016, ^13^Foster et al. 2021, ^14^Hensel et al. 2021.

Despite hundreds of ecological papers showing that biotic interactions are important to marine foundation species growth and success, we suggest that this knowledge could be better incorporated into coastal restoration planning, implementation, and evaluations of success. We argue that this is true whether animals are a direct goal of restoration, are an impediment to successful restoration, or provide functions that improve outcomes for habitat formers and the ecosystem. Several works describe these interactions within coastal ecosystems, such as mangrove forests (Gedan and Silliman 2009), saltmarshes (Derksen-Hooijberg et al. 2018), seagrass meadows (Valdez et al. 2020), coral reefs (Shaver and Silliman 2017, Seraphim et al. 2020), kelp forests (Eger et al. 2020), and oyster reefs (Reeves et al. 2020). Furthermore, although these general concepts are raised in restoration guidelines (e.g., McDonald et al. 2016, Morris et al. 2020, Eger et al. 2022, Shaver et al. 2022) and although we acknowledge that there are successful restoration projects in which animals have not been explicitly considered, a better understanding of when, why, and how to directly manipulate or support animals in restored habitats could significantly improve outcomes for many coastal restoration initiatives (Derksen-Hooijberg et al. 2018, Renzi et al. 2019, Gagnon et al. 2020).

## Restoration objectives and how animals can be incorporated into restoration actions

Coastal restoration efforts can have a range of different objectives that vary both in the importance of animals to meeting them and in the ways in which animals could be manipulated or supported to improve outcomes. For example, animals can be a direct or implicit goal of restoration (e.g., enhancing fisheries or improving habitat for a threatened species; Taylor et al. 2017), can be part of a more holistic goal to restore whole ecosystems (e.g., restoring habitats to reference conditions; McDonald et al. 2016), can provide functions that can benefit alternative goals of ecosystem restoration (Abelson et al. 2016, Gagnon et al. 2020, Valdez et al. 2020), or can be an impediment to restoration goals (e.g., overabundant grazers or invasive species; Morris et al. 2020). There is considerable risk that well-funded coastal restoration will be attempted globally with limited consideration of animals (Lee et al. 2019), subsequently limiting the success of many projects with a strong or sole habitat-forming species focus.

**Figure 2.**
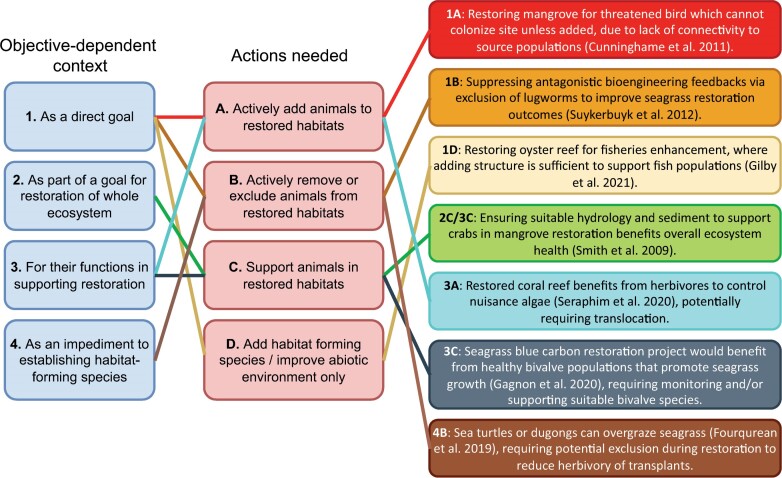
Four key contexts (1–4) in which animals could be manipulated and supported within coastal restoration, the actions (A–D) that can lead to positive outcomes, and examples to illustrate links between contexts and actions.

To encourage and guide scientists and managers to better incorporate animals into coastal restoration planning, implementation, and monitoring, we describe four key contexts in which animals could be manipulated and supported in restored habitats and the actions that can lead to positive outcomes (figure 2). We focus on six coastal marine ecosystems—mangrove forests, saltmarshes, seagrass meadows, coral reefs, macroalgae reefs, and oyster reefs—that have high intrinsic and extrinsic value to society, have unique ecological niches, are under accelerating threats from both land- and sea-based stressors, and are seeing a rapid rise in restoration initiatives (Perillo et al. 2018, Halpern et al. 2019, Bayraktarov et al. 2020). We outline a conceptual framework for an expanded approach that incorporates the benefits of a wider consideration of animals. Notwithstanding differences among restoration objectives, we argue that greater consideration of animals within planning, implementation, and monitoring can have benefits for most coastal restoration initiatives, and we use case studies to illustrate practical approaches (see box 1). Although there are important logistical, financial, legal, permitting, and societal considerations, because these are complex, involved, and require their own treatment to do them justice, we do not cover these in detail in the present article.

Box 1. **Case studies of manipulation or support of animals that did or could improve coastal restoration outcomes.**

***Adding clams during seagrass restoration in the United States (action A)***

**Figure 3.**
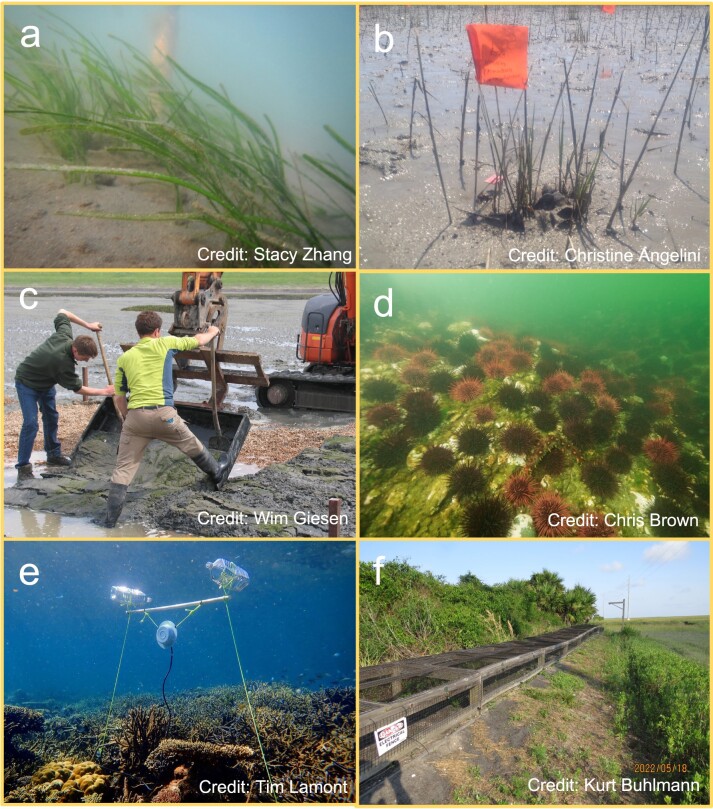
Images related to the six case studies: (a) clams added to restored seagrass patches, (b) mussels added into transplanted saltmarsh, (c) a crushed shell layer added underneath transplanted seagrass, (d) urchins requiring removal for macroalgae restoration, (e) healthy coral reef sounds played at degraded reefs, and (f) nesting mounds for terrapins added to saltmarsh. See the main text for case study descriptions and references.


Zhang and colleagues (2021) planted seagrass seeds within experimental plots (20 × 20 centimeter plots of *Zostera marina*) and added juvenile quahog clams (*Mercenaria mercenaria*; figure 3a). The addition of ten clams per plot led to significantly increased seagrass shoot length, a 500% expansion in patch size (versus no change in patches without clams), and ten times greater belowground biomass. Aboveground biomass and metrics related to seagrass reproduction—despite being several times higher in patches with clams—were not significantly different to control patches. The most likely casual mechanism was clams enhancing nitrogen availability. Zhang and colleagues (2021) also added harvest-size clams to 50 × 50 centimeter plots with transplanted adult seagrass (*Zostera marina* and *Halodule wrightii*), but clam addition had no effect.

***Adding mussels during saltmarsh restoration in the United States (action A)***

Derksen-Hooijberg and colleagues (2018) cotransplanted ribbed mussels (*Geukensia demissa*) into transplanted saltmarsh plots (*Spartina alterniflora*; 25 × 15 centimeter, diameter × depth) to evaluate the strategy for enhancing restoration success (figure 3b). Twenty mussels per plot increased saltmarsh vegetation growth and clonal expansion by 50%, as a result of mussels increasing nutrient levels and reducing sulfide stress. Following experimental vegetation removal that mimicked a disturbance event, vegetation in the plots containing mussels exhibited three times greater survival.

Box 1. Continued.

***Excluding lugworms during seagrass restoration in the Netherlands (action B)***

Creating physical barriers to separate animals that damage restored vegetation can improve establishment success. For example, Suykerbuyk and colleagues (2012) applied a crushed shell layer to shallow excavations underneath transplanted seagrass (*Zostera noltii*) to reduce interactions between seagrass and bioturbating lugworms (*Arenicola marina*; figure 3c). The shell layer reduced adult lugworm density by over 80%, and therefore reduced the lugworms’ negative engineering effects. This was predicted to be the primary driver of the 50%–140% enhancement in seagrass growth.

***Removing urchins during macroalgae restoration in Japan (action B)***

Herbivorous urchins can dominate rocky reefs and impede the restoration of macrolagal reefs, necessitating their removal (figure 3d; Miller and Shears 2022). Watanuki and colleagues (2010) employed citizen scientists to remove urchins (*Strongylocentrotus nudus*) in order to restore macroalgae beds (*Saccharina japonica* var. *religiosa* and *Undaria pinnatifida*). After 8 months, urchin densities were 0.1, 3.5, and 4.2 individuals per square meter for repeat removal, single removal, or no removal (control), respectively. Removal significantly enhanced kelp colonization and growth, with average standing macroalgae biomass within these treatments of 865, 150, and 0 grams per square meter, respectively.

***Supporting colonization of reef fishes using acoustic enrichment in Australia (action C)***

Larval fish can use acoustic cues when selecting habitats in which to recruit (Parmentier et al. 2015). Gordon and colleagues (2019) recorded noises from a healthy reef at night and evaluated the effects of playing these recordings on attracting fish to degraded reefs (figure 3e). Fish community development on acoustically enriched coral–rubble patch reefs was significantly enhanced across all major trophic guilds relative to acoustically unmanipulated controls, with doubled overall abundance and 50% greater species richness. Gordon and colleagues (2019) suggested that coupled with active restoration of coral reefs, acoustic attraction methods may expediate recovery processes.

***Building nesting mounds with protective boxes for terrapins in the United States (action C)***

Animals can be supported in restored habitats by the provision of artificial refuges. Quinn and colleagues (2015) built nesting mounds with protective boxes and electrified wire to reduce nest predation by raccoons and reduce road mortalities for diamond-backed terrapin (*Malaclemys terrapin*; figure 3f). The electrified wire significantly reduced predation rates, and excavated nest boxes afforded high rates of egg survivorship and hatching success, thereby supporting terrapin populations within saltmarsh and adjacent ecosystems. Deploying these within restored saltmarsh could therefore support the survival and reproduction of animals that are functionally important or of conservation concern.

### Actively add animals to restored habitats (action A)

The first action involves situations in which animals could be actively added into restored habitats. The *Field of Dreams* hypothesis, whereby restoring vegetation and physical structure are assumed to lead to animal colonization (i.e., “if you build it, they will come”) and that is sometimes applied to restoration projects, may not come to fruition; animals might not colonize restored habitats because of, for example, dispersal limitation or a lack of suitable source populations (Palmer et al. 1997, Lewis III 2010, Sundermann et al. 2011). In this instance, translocating animals into restored habitats may benefit coastal restoration outcomes, whether the animals are a direct goal (e.g., recovering populations of threatened birds that are incapable of colonizing restored habitats unassisted; figure 2, action 1A) or provide important functions that lead to improved restoration outcomes (e.g., algae herbivory or predation of herbivores that subsequently enhances the survival of the habitat-forming species; figure 2, action 3A; Seddon et al. 2014, Davis et al. 2019). Even when natural colonization is possible, assisting it through translocation may lead to substantial improvements in the recovery and development of habitat formers. For instance, transplanting mussels increased drought tolerance and vegetation growth by upward of 50% in restored saltmarsh (box 1; Angelini et al. 2016, Derksen-Hooijberg et al. 2018), incorporating sponges into coral reef restoration more than doubled successful coral colonization (Biggs 2013), and clam inclusion greatly enhanced seagrass biomass and meadow growth in seeding experiments (box 1; Zhang et al. 2021). Furthermore, adding animals to nursery rearing tanks with habitat formers being cultivated for outplanting can also improve outcomes (e.g., adding algal grazers to enhance reared coral survival; Neil et al. 2021).

Restocking of animals is a common tool in the management of nonmarine aquatic ecosystems, aimed at restoring water quality and vegetation characteristics (e.g., Angeler et al. 2003, Cowx and Gerdeaux 2004). Although less applied in the marine environment, some attempts at restocking have been carried out in marine ecosystems, mainly as a fishery management tool targeted at commercial fish populations (e.g., Lorenzen et al. 2010, Leber 2013). There have also been attempts at restocking invertebrate species such as the grazing gastropod *Trochus* sp. into coral reefs (e.g., Villanueva et al. 2010). Modeling suggests that restocking of grazing fish on coral reefs can facilitate reef recovery and can become profitable within several years (Obolski et al. 2016), and such an approach has been proposed to both significantly benefit the restoration of reef habitats and enhance fisheries stocks (Abelson et al. 2016). Ultimately, when appropriate and feasible, the active addition of animals that are the focus of restoration efforts or that help maintain vital ecological processes can enhance the success of coastal restoration initiatives (Swan et al. 2016).

### Actively remove or exclude animals from restored habitats (action B)

The second action involves situations in which animals could be actively removed or excluded from restored habitats. Under some circumstances animals can be detrimental to habitat-forming animals and, therefore, restoration outcomes, particularly early on as the habitat-forming species are becoming established (Poore et al. 2012). Targeted animal removal and exclusion can limit detrimental effects, such as impacts from bioturbation from worms (figure 2, action 1B, box 1; Suykerbuyk et al. 2012), overconsumption of planted seagrass by herbivores (figure 2, action 4B; Wendländer et al. 2019), overgrazing of macroalgae by urchins (Eger et al. 2022), and grazing and trampling of saltmarsh by ungulates (Davidson et al. 2017). Small-scale, manipulative experiments show that exclusion of herbivorous urchins and fish (Sharma et al. 2021), and corallivorous snails (Shaver et al. 2018) enhance coastal restoration outcomes via positive effects on habitat-forming species. The optimal intervention will likely depend on whether negative effects are expected or occurring, and the density dependence of those effects. For example, exclusion of urchins may only be necessary at the initial phases of planting macroalgae, until algal density reaches a point where positive density-dependent feedback processes within the population maintain its abundance (Eger et al. 2020). Although in many cases, the complete removal of the population (i.e., eradication) can be too costly and even impossible, suppression of the population to a point of “ecological eradication” may be sufficient under certain circumstances (*sensu* Green and Grosholz 2021).

### Supporting animals in restored habitats (action C)

The third action involves situations in which *supporting* animals (as opposed to actively adding animals; action A) can aid system recovery, enhance ecosystem resilience, and otherwise help meet restoration objectives. Designing restoration programs that identify and support key animal species and their functions can therefore benefit habitat formers and the restored system. Because animals use specific cues when selecting habitats (e.g., host plants, prey species, conspecifics, refuges) and require a suite of resources to persist in that habitat (e.g., sufficient prey resources; Van Dyck 2012), providing species-specific cues and resources can help assist the colonization and persistence of animals that are important for ecosystem function, persistence, and resilience. In addition to designing and modifying structural components of habitats to best support animals, animals can also be encouraged to colonize restored habitats through alternative means, such as with the playback of reef sounds to encourage fish colonization of coral reefs (see box 1; Gordon et al. 2019) or playing conspecific vocalizations to encourage bird colonization (Jones and Kress 2012). This requires knowledge of animal behavior and habitat requirements and of the species most important to improving restoration outcomes (Hale et al. 2020).

Supporting and attracting animals to benefit coastal restoration is not new; for decades, marine protected areas, for example, have been established in part to increase herbivore and predator abundance, which should, in turn, help with passive restoration of coral reefs (Topor et al. 2019) and kelp forests (Eger et al. 2020). Similarly, sea otter populations in the eastern North Pacific recovered dramatically following various conservation actions implemented decades ago, including restricting harvesting (Bodkin 2015). This recovery and the subsequent decrease in urchin populations has been important for the recovery of kelp at scale and may in fact be the preferred or most feasible action to passively restore habitat formers. Given animals can modulate ecosystem structure and function, similar acknowledgement needs to become the norm within active restorations (where management approaches such as distributing seeds, planting, and constructing habitats are implemented, as opposed to passive approaches that mitigate stressors preventing natural recovery; following Bayraktarov et al. 2016). In fact, where it is feasible, we argue that the explicit support of animals in restoration initiatives should be the rule rather than the exception. In addition, by taking a more targeted, animal-centric point of view *both* in design and in monitoring responses coastal restoration outcomes could be enhanced, even when the objective is not explicitly related to animals (e.g., blue carbon projects; figure 2, action 3C). One interesting avenue for future work with respect to supporting (or actively adding) animals should be to systematically assess whether a diverse suite of interactions from multiple animal species could best facilitate restoration, rather than focusing only a random or favorite one or two species.

### Add habitat-forming species or improve abiotic environment only (action D)

The fourth action involves situations in which animals are expected to colonize and persist in restored sites following the restoration of habitat-forming species. There are examples of successful coastal restoration efforts (with respect to creating functioning wildlife habitat) in which animals have not directly been manipulated nor habitats explicitly modified to support specific animal species per se (although success may still depend on healthy animal populations being able to colonize). For instance, deploying oyster reefs in Moreton Bay, Australia, led to rapid enhancement of fisheries species (figure 2, action 1D; Gilby et al. 2021); broadcasting seagrass seeds in Chesapeake Bay, in the United States, recovered diverse animal communities (Orth et al. 2020); and transplanting seagrass in California, in the United States, quickly recovered fish populations (Beheshti et al. 2021). Given the likelihood of animals being integral to the long-term health and resilience of restored habitats, ongoing animal monitoring where feasible is recommended and likely beneficial.

### Considerations, risks, and challenges

There are a series of important considerations, risks, and challenges when undertaking active interventions to directly manipulate or support animals. For instance, species translocations require sourcing individuals, which can be costly and ethically complex when removing individuals from wild populations (Pettorelli et al. 2018). Notably, many of the examples in this article involve supplementing already existing populations, such as the various bivalve species added to enhance seagrass or saltmarsh restoration (Derksen-Hooijberg et al. 2018, Zhang et al. 2021), with fewer risks relative to introducing new species. There is also a suite of challenges with releasing hatchery-reared animals that may, for example, perform worse in natural environments than wild conspecifics would, in part because of behavioral and cognitive differences (Lorenzen et al. 2013, Abelson et al. 2016, Näslund 2021). In addition, a detailed understanding of the system's ecology and robust predictions of the range of plausible outcomes are needed to help minimize the probability of or to manage unintended consequences (Sarrazin and Barbault 1996, Seddon et al. 2007). Adding or supporting animals can also lead to conflict with humans, such as the recovery of sea otter populations discussed above, which was unpopular in some regions because of otters competing with humans for harvested species such as clams, crabs, and urchins (Gregr et al. 2020). There are also important ethical considerations with excluding animals. The issue of turtles overgrazing seagrass restoration sites, for example, raises unaddressed questions about the ethics of turtle exclusion, because doing so may affect access to an important food resource for a threatened species and result in animal harm through starvation. There may also be community perception issues around culling animals, particularly if it involves native species, and culling (as per introduction) is likely to trigger a different set of permitting processes from the other restoration activities.

The various considerations, risks, and challenges are highly context specific and, in practice, should be evaluated in detail on a case-by-case basis. Previous works articulate questions that scientists and managers should answer or have some knowledge about prior to directly manipulating or supporting animals in restored habitats, such as those related to selecting source populations, the plausible implications for the wider ecosystem, and various ethical, permitting, and legal considerations (e.g., Sarrazin and Barbault 1996, Seddon et al. 2007, Armstrong and Seddon 2008, Houde et al. 2015, Nogués-Bravo et al. 2016, Berger-Tal et al. 2020). Resources also exist to guide practitioners with respect to translocations and species introductions, including general guidelines (e.g., IUCN 2013) and perspective reports with case studies (e.g., Soorae 2021), and these are also informative when attempting to attract and support animals. Ultimately, however, in many cases there are still gaps in our understanding of the ecology, which will lead to uncertainties in how and whether the manipulation or support of animals will influence the restoration trajectory. This requires ecological research as well as research on the cost-effectiveness of various actions.

## Applying structured decision-making to incorporate animals in restoration

We have argued for the benefits of explicit consideration of animals in restoration, and our argument is supported by a strong evidence base. One challenge now will be if, when, and how to scale up experimental restoration that has demonstrated how manipulating and supporting animals can aid restoration. A key impediment to explicitly including animals in restoration at scale will be demonstrating that the benefits exceed the costs (e.g., time, resources, ethical and legal requirements) and the risks (e.g., unintended impacts stemming from a lack of ecological knowledge on key processes, and uncertainty in outcomes). One useful approach for assessing the case for restoration is structured decision-making (SDM), a systematic and transparent approach to natural resource management. SDM is highly amenable to involving stakeholders in decision-making processes and is gaining traction in ecosystem restoration (Guerrero et al. 2017). SDM is based on decision theory and risk analysis and typically has seven key steps, as was articulated for kelp restoration in Gleason and colleagues (2021). We show how the SDM framework can apply to the question of whether to explicitly include or exclude dependent fauna into restoration of habitat-forming marine and coastal species using three case studies in table 1. This table is intended to be indicative and would require further development to guide restoration science and practice. In reality, applying SDM to restoration requires greater detail, a more holistic understanding of the system, and input from various stakeholders, and ultimately, the best approach may involve a combination of actions (e.g., Gleason et al. 2021).

**Table 1.** Applying a simplified structured decision-making (SDM) process to three hypothetical case studies with varying objective-dependent animal roles.

Key steps	Hypothetical case studies		
1. Problem formulation	1. Restore degraded saltmarsh to reference condition.	2. Enhance fisheries via coral reef restoration.	3. Expand seagrass area for carbon sequestration.
2. Set clear objectives	Reach similar floral and faunal species richness to reference sites within 7 years.	Increase fisheries productivity by 30% within 10 years.	Double carbon stock within 15 years.
3. Identify actions, including parameterization of costs and likelihoods of achieving objectives	A: Translocate supporting animals (e.g., bivalves) B: Plant saltmarsh C: Remove exotic or damaging species (e.g., ungulates)	A: Translocate corals B: Breed and release juvenile fisheries species C: Translocate supporting animals (e.g., algae grazing gastropods)	A: Plant seagrass B: Translocate supporting animals (e.g., bivalves, algae grazers) C: Exclude hindering animals (e.g., herbivores)
4. Estimate consequences. This text articulates the prediction of outcomes for one of the actions identified in step 3. A similar approach would be taken for all actions, and the estimates used to inform 5.	Generate predictions for outcomes of actions A, B, and C. For example, using inferences from studies on mussel translocations (e.g., Derksen-Hooijberg et al. 2018) and meta-analyses that compare animal populations between restored and reference ecosystems (e.g., Sievers et al. 2018). For action A, we predict the benefits of translocating mussels at various densities on drought tolerance and growth of saltmarsh vegetation, and relate this to biodiversity benefits. Hypothetical prediction for A: Richness surpasses 80% of reference levels within a 7-year timeframe.	Generate predictions for outcomes of actions A, B, and C. E.g., using numerical fisheries models that predict fish production from coral reef condition (Rogers et al. 2018). For action C, we predict the benefits of translocating algal grazers to the survival and growth of newly transplanted corals, and the subsequent outcomes for fish production. Hypothetical prediction for C: Grazers will enhance coral survival by 30%–50%, leading to a 2–3 tons per hectare increase in fish biomass after 10 years.	Generate predictions for outcomes of actions A, B, and C. E.g., using models that predict CO_2_ capture from restored seagrass extent (e.g., Duarte et al. 2013b). For action C, we predict the exclusion of herbivores promotes seagrass growth (e.g., Burkholder et al. 2013) and, we can link this to predicted carbon sequestration and stocks. Hypothetical prediction for C: Exclusion fences will eliminate grazing by turtles, tripling seagrass biomass, and leading to a doubling of carbon stock within 15 years.
5. Evaluate trade-offs	Evaluate trade-offs across alternative actions from 3 and 4 to determine which one or more best meets that objective (dependent on importance, cost, benefit, degree of certainty, risk, constraints, etc.).		
6. Make decisions	Make decision on the basis of the information gained in steps 3–5, by assessing which options are most likely to achieve the desired goals and objectives set out in steps 1 and 2 within the constraints of the project (budget, time, feasibility, etc.).		
7. Act, monitor and learn	A: Given the ease at which manipulations occur, conduct replicated experiment to examine the effect of mussel addition (and density effects) on marsh growth and survival. Where possible, extend monitoring to other species in the food web. Measure biodiversity across restored and reference sites.	C: Monitor grazers, algal growth, and coral growth and survival. Contrast outcomes with unmanipulated areas. Develop models to identify optimal grazer densities (both densities the system can support, and those that maintain coral survival and growth). Continue to monitor fisheries productivity.	C: Monitor seagrass growth and survival. Once seagrass is established, remove cages to allow potential positive species interactions. Maintain monitoring; if overgrazing continues, refencing may be needed. Quantify carbon stock across natural and restored seagrass meadows.

*Note:* The SDM approach was based on steps in Gleason and colleagues (2021).

## Conclusions

Ongoing destruction and degradation of coastal habitats and the subsequent loss of service benefits to people have necessitated accelerating restoration efforts. But restoration without animals may not achieve the desired outcome. Although there are many impediments to effective coastal restoration, identifying when, why, and how to directly manipulate or support animals can lead to substantial improvements in outcomes for habitat-forming species and ecosystem services. By outlining how animals play important roles across different restoration objectives, articulating key contexts in which animals can be explicitly incorporated in coastal restoration, and illustrating these ideas with practical case studies, we hope to encourage scientists and managers to better incorporate animals into coastal restoration planning, implementation, and monitoring.

## Author Biographical

Michael Sievers (m.sievers@griffith.edu.au), Christopher J. Brown (chris.brown@griffith.edu.au), Christina A. Buelow (c.buelow@griffith.edu.au), Andria Ostrowski (andria.ostrowski@griffithuni.edu.au), Mischa P. Turschwell (m.turschwell@griffith.edu.au), and Rod M. Connolly (r.connolly@griffith.edu.au) are affiliated with the Coastal and Marine Research Centre, the Australian Rivers Institute, and with the School of Environment and Science at Griffith University, in Gold Coast, Queensland, Australia. Robin Hale (rob.hale@delwp.vic.gov.au) is affiliated with the Arthur Rylah Institute for Environmental Research at the Department of Environment, Land, Water, and Planning, in Heidelberg, Victoria, Australia. Megan I. Saunders (megan.saunders@csiro.au) is affiliated with CSIRO Oceans and Atmosphere, in St Lucia, Brisbane, Australia. Brian R. Silliman (brian.silliman@duke.edu) and Stephanie R. Valdez (stephanie.valdez@duke.edu) are affiliated with the Nicholas School of the Environment at Duke University, in Beaufort, North Carolina, in the United States. Stephen E. Swearer (s.swearer@unimelb.edu.au) is affiliated with the National Centre for Coasts and Climate and with the School of Biosciences at the University of Melbourne, in Parkville, Victoria, Australia.
